# PI4 Kinase Is a Prophylactic but Not Radical Curative Target in Plasmodium vivax-Type Malaria Parasites

**DOI:** 10.1128/AAC.03080-15

**Published:** 2016-04-22

**Authors:** Anne-Marie Zeeman, Suresh B. Lakshminarayana, Nicole van der Werff, Els J. Klooster, Annemarie Voorberg-van der Wel, Ravinder R. Kondreddi, Christophe Bodenreider, Oliver Simon, Robert Sauerwein, Bryan K. S. Yeung, Thierry T. Diagana, Clemens H. M. Kocken

**Affiliations:** a Biomedical Primate Research Centre, Department of Parasitology, Rijswijk, The Netherlands; b Novartis Institute for Tropical Diseases, Singapore, Singapore; c Medical Parasitology, Radboud University Nijmegen Medical Centre, Nijmegen, The Netherlands

## Abstract

Two Plasmodium PI4 kinase (PI4K) inhibitors, KDU691 and LMV599, were selected for *in vivo* testing as causal prophylactic and radical-cure agents for Plasmodium cynomolgi sporozoite-infected rhesus macaques, based on their *in vitro* activity against liver stages. Animals were infected with P. cynomolgi sporozoites, and compounds were dosed orally. Both the KDU691 and LMV599 compounds were fully protective when administered prophylactically, and the more potent compound LMV599 achieved protection as a single oral dose of 25 mg/kg of body weight. In contrast, when tested for radical cure, five daily doses of 20 mg/kg of KDU691 or 25 mg/kg of LMV599 did not prevent relapse, as all animals experienced a secondary infection due to the reactivation of hypnozoites in the liver. Pharmacokinetic data show that LMV599 achieved plasma exposure that was sufficient to achieve efficacy based on our *in vitro* data. These findings indicate that Plasmodium PI4K is a potential drug target for malaria prophylaxis but not radical cure. Longer *in vitro* culture systems will be required to assess these compounds' activity on established hypnozoites and predict radical cure *in vivo*.

## INTRODUCTION

New malaria drugs are desperately needed as malaria parasites become more resistant to current treatments. The spread of artemisinin resistance threatens 200 million to 300 million malaria patients per year, primarily in sub-Saharan Africa (1). Despite significant efforts in recent years toward the development of new drugs against falciparum malaria, efforts for Plasmodium vivax, the predominant malaria species outside Africa, have not enjoyed the same level of focus as Plasmodium falciparum. Patients infected with P. vivax malaria require a special drug treatment regimen to achieve complete cure (2), due mainly to the parasite's ability to form dormant liver-stage parasites. These dormant forms (hypnozoites) can reactivate months or years later to give rise to new symptomatic blood-stage infections without being exposed to a new infection by mosquitoes (3). These relapses stem from a hidden parasite reservoir in the host liver and form an additional complication for the treatment and cure of P. vivax patients. Currently, only one registered drug, primaquine (PQ), can be prescribed to treat hypnozoites and thus provide a radical cure for vivax malaria (4). However, PQ is contraindicated in glucose-6-phosphate dehydrogenase-deficient individuals because of potentially severe blood toxicity (5), which is the primary limitation of this and other 8-aminoquinoline-derived drugs. To address emerging parasite resistance toward PQ (6, 7), new and safer non-8-aminoquinoline drugs for radical cure are needed.

We previously described an *in vitro* drug screening platform for the liver stages of the primate malaria species Plasmodium cynomolgi, which shares the same characteristic hypnozoite biology as P. vivax malaria (8, 9). We identified one compound, KAI407, from a new chemical class which displayed an activity profile similar to that of PQ in its ability to clear existing parasite liver stages *in vitro* (10). The target of KAI407 was later determined to be Plasmodium PI4 kinase (PI4K) (11), and we hypothesized that compounds that are active against this target may also possess antihypnozoite activity *in vivo* similar that of PQ. We have since identified other analogues, KDU691 (12) and LMV599 (our unpublished data), which have improved potency on P. cynomolgi liver stages and better drug-like properties than KAI407. We present the *in vivo* efficacy profiles of these inhibitors when orally administered as causal prophylaxis or radical-cure agents in P. cynomolgi sporozoite-infected rhesus monkeys. The results of these experiments were used to determine if Plasmodium PI4 kinase is a potential target for P. vivax radical cure as well as to validate the *in vitro* screening platform to identify new antihypnozoite drug leads.

## MATERIALS AND METHODS

Detailed materials and methods are provided in the supplemental material.

### Animal experiments.

For *in vivo* testing of KDU691, LMV599, and PQ as prophylaxis and/or radical-cure agents, we used four experimental rhesus monkeys for each dosing group (power calculation in Table S1 in the supplemental material), the one exception being LMV599, where three animals were used to evaluate its prophylactic activity.

All rhesus macaques (Macaca mulatta) used in this study were captive bred for research purposes and were housed at Biomedical Primate Research Centre (BPRC) facilities in compliance with the Dutch law on animal experiments; European directive 2010/63/EU; and the standard for humane care and use of laboratory animals by foreign institutions, identification number A5539-01, provided by the Department of Health and Human Services of the U.S. National Institutes of Health (NIH). The BPRC is an AAALAC-certified institute. Prior to the start of monkey experiments, protocols were approved by the local independent ethical committee, according to Dutch law.

The Institutional Animal Care and Use Committee (IACUC) of Novartis Institute for Tropical Diseases (NITD), registered with the Agri-Food and Veterinary Authority (AVA), Government of Singapore, reviewed and approved all animal experimental protocols, including the mouse experiments.

### Generation of P. cynomolgi sporozoites.

For each batch of sporozoites required, one rhesus macaque was infected with blood-stage parasites, and mosquitoes were fed at the appropriate time point and monitored for infection rates (10). Sporozoites were harvested from P. cynomolgi-infected mosquitoes ∼16 days after ingestion of the infected blood meal.

### Compound synthesis.

KDU691 was prepared as previously described (12). The synthesis and optimization of LMV599 will be described in a separate manuscript (our unpublished data).

### *In vitro* liver-stage drug assays with P. cynomolgi.

*In vitro* infections of primary rhesus hepatocytes with P. cynomolgi sporozoites were performed according to methods described previously by Zeeman et al. (10). At day 6 postinfection (p.i.), the assay mixtures were fixed and stained with anti-P. cynomolgi Hsp70 rabbit antiserum and a fluorescein isothiocyanate (FITC)-labeled secondary antibody (goat anti-rabbit). Plates were analyzed with the Operetta high-content imaging system, differentially counting hypnozoites and developing extraerythrocytic forms (EEFs), based on parasite size (10).

### Sporozoite infection and drug treatment of rhesus macaques.

Sporozoites were harvested from P. cynomolgi-infected mosquitoes, washed with phosphate-buffered saline (PBS), and diluted to 100,000 sporozoites (spz)/ml in PBS. One-milliliter aliquots of sporozoites were prepared and injected into monkeys via intravenous (i.v.) injection.

Monkeys were treated according to the schedule shown in Table 1 (stratification of treatment groups is given in the supplemental material). During treatment, monkeys were weighed daily and received the compound via gavage, followed by gastric feeding (weight change during treatment is shown in Table S6 in the supplemental material). Monkeys in the prophylaxis groups (groups 691-proph and 599-proph) were treated ∼20 min after the i.v. sporozoite injection. The other monkeys were treated when all the monkeys in the experiment (except those in the prophylaxis group) were blood-stage patent (at day 11 p.i.). To kill the blood-stage parasites, all monkeys received a 5-day treatment of 7.5 mg/kg of body weight of chloroquine (CQ) (intramuscular [i.m.] injection), during compound dosing. KDU691 was formulated in 0.5% methylcellulose and 0.5% Tween 80 in water. LMV599 was formulated as a solid dispersion in a solution containing 5.6% (wt/wt) Tween 80–44.4% hydroxypropyl methylcellulose (HPMC) E3–38.9% Soluplus–11.1% vitamin E d-alphatocopheryl polyethylene glycol 1000 succinate (TPGS).

**TABLE 1:** Schedule for dosing of treatment groups^*a*^

Group	No. of animals	Compound	Amt of compound (mg/kg)	Carrier^*b*^	First day of dosing (day p.i.)	Duration of dosing (days)
691-C	4	None			11	5
691-proph	4	KDU691	20	1	0	5
691-RC	4	KDU691	20	1	11	5
599-C	4	None		2	11	5
599-proph	3	LMV599	25	2	0	1
599-RC	4	LMV599	25	2	11	5
PQ-RC	4	PQ	1.8	3	11	5

a Two independent *in vivo* experiments were performed. The first experiment was performed with KDU691 as both prophylaxis and radical cure. Monkeys of group 691-C (control group) were infected together with the 691 treatment groups. The second *in vivo* experiment was performed with the optimized product LMV599. Monkeys of group 599-C (control group) were infected together with the 599 radical-cure treatment group and the PQ treatment group. In the prophylaxis groups, the drug was administered 20 min after i.v. sporozoite injection.

b Carrier 1 contained 0.5% methylcellulose–0.5% Tween in water, carrier 2 was a 5.6% (wt/wt) Tween 80–44.4% HPMC E3–38.9% Soluplus–11.1% vitamin E TPGS solid dispersion, and carrier 3 contained 15% syrup in tap water.

Blood samples were collected for clinical evaluation and pharmacokinetic (PK) analysis (see Table S2 in the supplemental material).

### Monitoring parasitemia.

Parasitemia was closely monitored by thin-film analysis. Animals were trained to present for thigh pricks, which was followed by a reward. Thin-film slides were stained with 20% Giemsa stain, and at least 50 fields (±20,000 erythrocytes [RBCs]) were analyzed with a bright-field microscope (×1,200 magnification). As soon as all monkeys other than the prophylactic treatment groups were blood-stage positive, treatment was started as indicated above (Table 1). After the last day of compound dosing, the absence of blood-stage parasites was confirmed by thin smears. From day 23 p.i., monkeys were monitored daily for relapses. The KDU691 radical-cure (group 691-RC) treatment and control groups were monitored until the first relapse. The LMV599 control (group 599-C), radical-cure (group 599-RC), and PQ radical-cure (group PQ-RC) treatment groups were monitored until the second relapse or until day 100 p.i. (whichever came first). At the end of the study, monkeys that had relapsed received a 14-day primaquine treatment, with a final check 2 weeks after the last primaquine dose.

The prophylaxis groups were monitored daily until day 22 p.i. From day 22 to day 45 p.i., they were monitored three times a week, and from day 45 to day 105 p.i. (100 days after treatment), they were monitored twice a week.

### *In vivo* PK studies of KDU691 and LMV599 in CD-1 mice and monkeys.

Details of *in vivo* PK studies of KDU691 and LMV599 in CD-1 mice and monkeys can be found in the supplemental material.

### Statistical analysis of data.

Statistical analyses were performed by using the R language and environment for statistical computing (R Foundation for Statistical Computing, Vienna, Austria [see http://www.R-project.org/]). Survival data (time to patency) were evaluated by using the log rank test, where the different treatment groups were individually compared to the placebo control group.

## RESULTS

### Selection of KDU691 for *in vivo* testing.

**FIG 1:**
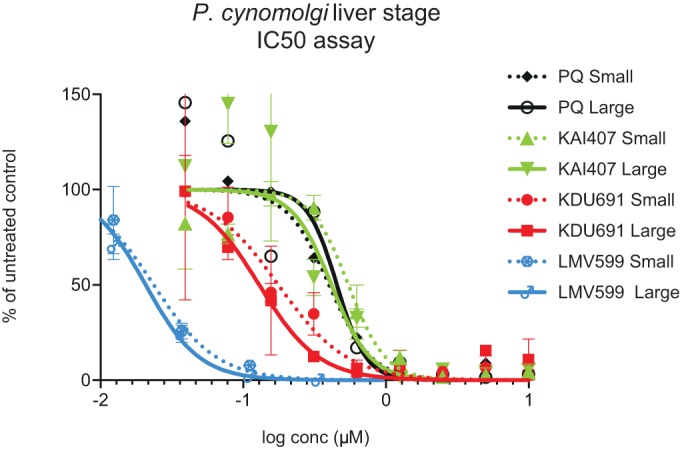
*In vitro* assays with KDU691 and LMV599. *In vitro* activity against P. cynomolgi liver stages was determined for both compounds. The numbers of small (hypnozoites) and large (developing EEFs) parasites were determined by using Operetta and image analysis of primary rhesus hepatocyte cultures infected with P. cynomolgi sporozoites and exposed to the compound for 6 days (10). KDU691 and LMV599 were more active than primaquine and KAI407 (the lead compound) in the assay. IC_50_s were determined by using the least-squares nonlinear regression method (Prism) for small and large parasites. Data from several independent experiments were used to calculate the IC_50_s for the two compounds. Results of one representative experiment are shown. The IC_50_s against hypnozoites were 0.18 μM for KDU691 (range, 0.06 to 0.7 μM) and 18 nM for LMV599 (range, 3.5 to 47 nM). The IC_50_s against developing EEFs were 0.06 μM for KDU691 (range, 0.02 to 0.4 μM) and 12 nM for LMV599 (range, 7 to 21 nM). IC_50_s against hypnozoites were 0.69 μM (0.84 to 0.55 μM) for KAI407 and 0.84 μM (0.29 to 2.31 μM) for PQ. IC_50_s against developing EEFs were 0.64 μM (0.86 to 0.42 μM) for KAI407 and 0.37 μM (3.27 to 0.33 μM) for PQ (10).

KDU691 displayed improved activity over both PQ and the lead compound KAI407 in the *in vitro* liver-stage assay (Fig. 1). KDU691 had 50% inhibitory concentrations (IC_50_s) of 0.18 ± 0.21 μM against hypnozoite forms and 0.061 ± 0.048 μM against liver schizonts (means of data from 9 independent assays). Infection with Plasmodium berghei luciferase sporozoites followed by a single dose of 7.5 mg/kg KDU691 gave complete protection in mice (12), showing that this class of compounds displays excellent causal prophylactic activity. Pharmacokinetic analysis in mice and monkeys showed good oral bioavailability (52 to 66%) and compound partitioning in the liver (1.7× for the maximum concentration of drug in serum [*C*_max_] and 3.1× for exposure over plasma concentrations).

### *In vivo* evaluation of KDU691: prophylactic treatment with KDU691 completely blocks development of blood-stage parasitemia.

During the 5 days of dosing, no major weight changes were observed in the animals that received KDU691 as prophylactic treatment (group 691-proph) (see Table S5 in the supplemental material). PK analysis was performed on blood samples taken on the first and last days of dosing (see Table 3). From the fourth day of dosing, the animals that were treated with KDU691 showed a transient yellow skin color. This side effect was the result of an accumulation of endogenous bilirubin and was previously observed in rats. Although bilirubin levels and skin coloration resolved 2 days after the last dose of KDU691, we opted to measure liver enzyme and bilirubin levels in the radical-cure groups to monitor potential side effects.

Other than this observation, the group 691-proph animals remained blood-stage negative throughout the experiment (until day 102 p.i.), demonstrating that the causal prophylactic treatment with KDU691 was completely effective (Table 2; see also Fig. S1A in the supplemental material). All animals from the untreated control and radical-cure groups became positive for primary blood-stage parasites.

**TABLE 2:** Days to patency and first and second relapses for all treatment groups^*a*^

Group	Monkey	Treatment	Day p.i. to primary parasitemia	Day p.i. to first relapse	Day p.i. to second relapse
691-C	1	None	8	25	—
	2		9	26	—
	3		9	23	—
	4		9	26	—
691-proph	5	Prophylaxis with 20 mg/kg KDU691	ND		
	6		ND		
	7		ND		
	8		ND		
691-RC	9	Radical cure with 20 mg/kg KDU691	9	31	—
	10		9	32	—
	11		9	32	—
	12		9	32	—
599-C	13	None	10	33	65
	14		10	31	50
	15		9	28	44
	16		10	28	44
599-proph	17	Single-dose prophylaxis with 25 mg/kg LMV599	ND		
	18		ND		
	19		ND		
599-RC	20	Radical cure with 25 mg/kg LMV599	10	26	45
	21		10	27	44
	22		10	26	46
	23		10	27	45
PQ-RC	24	Radical cure with 1.8 mg/kg PQ	9	ND	ND
	25		10	ND	ND
	26		10	47	100
	27		11	68	ND

a Individual data are shown for each animal. —, no data were collected; ND, not detected. Monkeys of group 691-C (control group) were infected together with the 691 treatment groups, and monkeys of group 599-C were infected together with the 599 radical-cure treatment group and the PQ treatment group. Monitoring for blood-stage parasites was done by thin-film analysis. In each smear, at least 50 fields (>20,000 RBCs) were analyzed for the presence of blood-stage parasites at a ×1,200 magnification. A short delay to relapse of 6.8 days was seen in the 691-RC group, which was significant (*P* value of 0.00912 according to a log rank test). No blood-stage parasites were found in any of the prophylaxis arms. Neither of the compounds completely blocked the occurrence of a relapse.

### Primary blood-stage parasitemia development after sporozoite infection.

From day 8 onward, all animals that were not in the 691-proph group were monitored for blood-stage parasites. The first blood-stage parasites became apparent after 8.9 ± 0.35 days (averaged over all animals; range, 8 to 9 days) (Table 2; see also Fig. S1A in the supplemental material). The monkey that was the last to receive the sporozoite injection became blood-stage positive at day 9 p.i. The radical-cure treatment was started at day 11 p.i. (Table 1), and the weight of the animals did not change significantly during treatment (see Table S5 in the supplemental material).

### Monitoring relapse after KDU691 radical cure.

After the last day of treatment, a blood smear was taken to verify that the animals were cured from blood-stage parasites. All animals were blood-stage negative at day 18 p.i. Relapse of the control group was detected at 25 ± 1.4 days p.i. (range, 23 to 26 days). The KDU691 radical-cure group (group 691-RC) became blood-stage positive again at 31.8 ± 0.5 days p.i. (range, 31 to 32 days) (Table 2; see also Fig. S1B in the supplemental material).

The 691-RC group showed a delay to relapse of 6.8 days compared to the control group. This result was statistically significant (X^2^ = 6.8; df = 1; *P* = 0.00912 [as determined by a log rank test]), but none of the animals treated with KDU691 were radically cured.

### Bilirubin levels and liver function.

**FIG 2:**
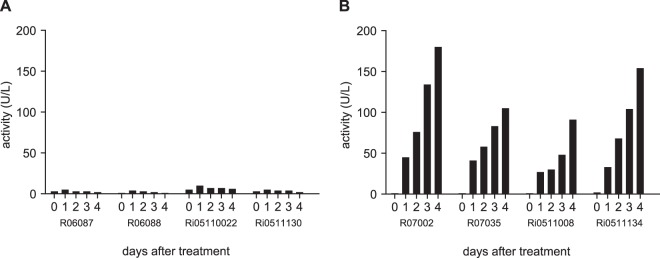
Bilirubin accumulation in group 691-RC animals. Clinical chemistry samples were taken daily from group 691-RC (radical cure) animals during treatment. One clinical chemistry parameter was significantly different in the treatment groups: bilirubin. (A) Bilirubin levels in group 691-C animals remained normal. (B) Bilirubin in group 691-RC monkeys (20 mg/kg KDU691 per day during 5 days) accumulated over time. Other clinical chemistry measurements were not significantly different between the control and treatment groups.

Four days after the start of treatment of the group 691-proph monkeys, the monkeys developed a yellow skin color, especially in the facial and chest areas. The discoloration disappeared spontaneously ∼5 days after the last dose. The same was observed for the monkeys in the 691-RC group (which were treated 11 days later). Clinical chemistry analysis of the group 691-RC monkeys revealed that bilirubin levels accumulated during the 5-day radical-cure treatment with KDU691 (Fig. 2). Other clinical parameters did not change significantly from the control group values.

### Selection of LMV599 and shortened PQ treatment for a follow-up *in vivo* efficacy experiment.

The 6.8-day delay to first relapse observed in the 691-RC group suggested that only part of the hypnozoite population was eliminated. We hypothesized that improving the *in vitro* potency as well as increasing the plasma exposure might lead to a longer delay to relapse. LMV599 was identified as the follow-up compound to KDU691 based on these criteria (Fig. 1).

To test whether a statistically significant delay to relapse would also be biologically relevant, we included a shortened PQ treatment arm in parallel to the 5-day treatment course with LMV599. This shorter treatment regimen was recently reported to completely cure toque monkeys from P. cynomolgi infections (13) and would be an important refinement in the P. cynomolgi-rhesus model. Animals not achieving radical cure after 5-day PQ treatment but displaying a delay to relapse would indicate a depletion of hypnozoites (14). In comparison, a significant delay to relapse for PI4K inhibitors, similar to that observed with a 5-day PQ treatment, would suggest some level of antihypnozoite activity *in vivo*.

### Single-dose prophylaxis with LMV599.

To confirm the prophylactic efficacy of LMV599, we designed a single-dose study in comparison to the above-described multiple-dose KDU691 experiment. Three monkeys were infected with 100,000 spz (sporozoites were shown to be infectious *in vitro*) and treated with a single oral dose of 25 mg/kg of LMV599, 20 min after infection. Blood plasma analysis of animals showed good compound exposure at >3 times the IC_50_ on small forms for 12 h (Table 3), and animals were monitored daily for patency until day 22 p.i. and then three times a week until day 45 p.i. and twice a week until day 100 p.i. None of the dosed animals exhibited detectable blood-stage parasitemia throughout the observation period, demonstrating that LMV599 has single-dose prophylactic activity against P. vivax-type parasites (Table 2; see also Fig. S2A in the supplemental material).

**TABLE 3:** PK-PD parameters for KDU691 and LMV599 in preclinical species^*a*^

Compound	Species	Dose (mg/kg)	Oral PK parameter			PK-PD index (free)		
			*C*_max_ (μM)	AUC (μM · h)	*F* (%)	f*C*_max_/TRE	fAUC/TRE	% f*T* > TRE
KDU691	Mouse	25	20.51	30.56	60			
		25^*b*^	34.77	94.22				
	Monkey	20	4.31	38.82		1.45	13.08	16.7
LMV599	Mouse	2.5	1.11	3.92	30			
		2.5^*b*^	3.67	17.53				
	Monkey	25^*c*^	2.59	15.38		11.99	71.20	50
		25^*d*^	1.49	10.82		6.90	50.09	50

a AUC, area under the concentration-time curve from 0 to 24 h; *F*, oral bioavailability, *C*_max_/TRE, ratio of *C*_max_ to the threshold (TRE = 3× the IC_50_); AUC/TRE, ratio of the area under the concentration-time curve from 0 to 24 h to the threshold; % *T* > TRE, percentage of the 24-h period during which the free compound concentration exceeded the threshold; f, free fraction (based on monkey plasma protein binding [81.8% and 75% for KDU691 and LMV599, respectively]).

b Liver PK.

c Causal prophylactic study.

d Radical-cure study.

### Five-day radical-cure treatment with LMV599 or PQ.

Twelve animals were selected for this study, stratified (see Table S4 in the supplemental material), and infected with 100,000 spz i.v. The first blood-stage parasites became apparent after 9.9 ± 0.5 days (range, 9 to 11 days). Treatment was started at day 11 p.i., when all animals were blood-stage positive (Table 1). Animals did not show significant weight change during the dosing period (see Table S5 in the supplemental material). Clinical chemistry and hematology analyses showed increased activity of the liver enzymes alkaline transaminase (*P* value of 0.0269) and gamma glutamyl transpeptidase (*P* value of 0.0409) (see Table S6 in the supplemental material) in LMV599-treated animals. Other clinical and hematological parameters, including bilirubin, did not differ significantly between control and LMV599-treated animals.

### Monitoring the first relapse after LMV599 or PQ radical cure.

After the last day of treatment, a blood smear was taken to verify the absence of blood-stage parasites. The first relapse of the control group was detected at 30 ± 2.5 days p.i. (range, 28 to 33 days). The LMV599 radical-cure group (group 599-RC) became blood-stage positive at 26.5 ± 0.6 days p.i. (range, 26 to 27 days) (Table 2; see also Fig. S2B in the supplemental material). In the PQ-treated group, only two of the four animals relapsed at day 57.5 ± 14.8 p.i. (one at day 48 p.i. and one at day 65 p.i.), and the other two PQ-treated monkeys did not relapse during the time of monitoring (up to 100 days p.i.) and were deemed fully cured (Table 2; see also Fig. S2C in the supplemental material).

### Monitoring the second relapse after LMV599 or PQ radical cure.

Animals that relapsed were treated with an additional 5-day course of CQ, starting the day after the positive blood smear was obtained. The absence of blood-stage parasites was confirmed at the end of CQ treatment, and the monkeys were monitored again from day 8 after the last CQ dose until they became blood-stage positive or until day 100 p.i. The control group monkeys had the second relapse at 51 ± 15 days p.i. (range, 44 to 65 days), while the LMV599-treated animals relapsed at 45 ± 0.8 days (range, 44 to 46 days) after sporozoite infection. In the PQ treatment group, one monkey relapsed for the second time, at day 100 p.i., while the other PQ-treated monkey did not relapse for a second time during the 100 days that it was monitored (Table 2; see also Fig. S2D in the supplemental material).

The times to first and second relapses were not significantly different between the LMV599-treated group and the untreated control group, while the 5-day PQ-treated group resulted in the radical cure of two out of four animals and a significant delay to first relapse (*P* value of 0.000237) in the other two animals.

### PK-PD of LMV599 in 5-day radical-cure treatment.

Pharmacokinetic analysis in mice showed good exposure and compound partitioning in the liver (3.3 and 4.5 times the *C*_max_ and exposure over plasma concentrations, respectively).

At a comparable dose in monkeys, both the *C*_max_ and exposure were 2-fold lower for LMV599 than for KDU691 (Table 3). However, because of the improved *in vitro* potency on P. cynomolgi liver stages (∼10-fold), LMV599 displayed good PK-pharmacodynamic (PD) indices and coverage (*T* > TRE [24-h period during which the free compound concentration exceeded the threshold]), which were markedly improved over KDU691 indices, meaning that LMV599 was predicted to have better exposure and plasma concentrations over 3 times the IC_50_ during the 5 days of dosing. Despite the improvements in the LMV599 PK-PD indices, radical cure was not achieved, and there was no delay to relapse after LMV599 treatment.

## DISCUSSION

Using an *in vitro* screening assay, we identified the Plasmodium PI4K inhibitor KAI407 as an active compound against P. cynomolgi hypnozoites (10). Chemical optimization of this compound led to KDU691, with improved potency in the *in vitro* assay and better drug-like properties (12). To confirm the antihypnozoite activity observed in the *in vitro* assay, KDU691 was evaluated for *in vivo* activity in rhesus macaques infected with P. cynomolgi sporozoites. The prophylactically treated monkeys failed to become blood smear positive, while untreated control animals showed normal parasitemia development (primary parasitemia at day 8.9 p.i., with the first relapse within a month). Thus, 5-day oral treatment with 20 mg/kg of KDU691 shortly after infection eradicated all liver-stage parasites, including hypnozoites, and its activity is consistent with the *in vitro* activity profile of KDU691 against P. cynomolgi liver stages. However, animals dosed with KDU691 in a radical-cure mode relapsed only 6.8 days later than the control group monkeys.

We surmised that KDU691 treatment only partially cleared the hypnozoite population and that the delay to relapse could be extended (>6 days) with a superior compound with improved potency and plasma exposure *in vivo*.

A short delay to relapse can also be explained if the compound suppressed the development of liver-stage parasites only over the course of drug treatment (5 days), which resume normal development in the absence of drug pressure. This would also result in a delay to relapse about equal to the time of drug treatment and similar to the observed delay to relapse. In this case, a second relapse should not be influenced by the treatment. Hence, we also monitored the monkeys up to the second relapse in the subsequent experiment with LMV599.

Chemical optimization of KDU691 resulted in LMV599, which was 10-fold more potent than KDU691 in the *in vitro* antihypnozoite assay and had improved PK-PD indices. Indeed, a single dose of 25 mg/kg LMV599 completely prevented the development of blood-stage parasitemia in three out of three monkeys, indicating its effectiveness in blocking parasite development in the liver and preventing blood-stage patency. Unfortunately, in the radical-cure experiment, animals treated with LMV599 did not show any significant delay to the first or second relapse compared to untreated controls, despite the improved PK-PD indices compared to those of KDU691. It is possible that the threshold chosen (3 times the IC_50_) underestimated the plasma concentrations required for efficacy *in vivo* and that a more stringent threshold, such as 2 times the IC_99_, would better predict the effective drug concentration levels achieved under the *in vitro* assay conditions. In comparison, of the four animals treated with a suboptimal dose of PQ, two were completely cured, while the two animals that relapsed had a much longer delay to relapse than observed in the KDU691 experiment. We conclude that genuine radical curative test compounds in this animal model, even at suboptimal doses, would yield complete cure and/or a long delay to relapse, consistent with what was previously observed by Schmidt et al. (14).

Collectively, these data provide evidence that Plasmodium PI4K is a potential target for prophylaxis for malaria but not for radical cure.

PI4K inhibitors were the first compounds identified in the P. cynomolgi *in vitro* assay to have antihypnozoite activity similar to that of PQ (10). This study, however, has shown that the *in vitro* activity against hypnozoites did not translate to *in vivo* radical-cure activity. There is a possibility that the *in vitro* assay format, i.e., drug exposure from days 0 to 6 post-hepatocyte infection, has a bias for prophylactic rather than radical curative activity. Drug treatment in the P. cynomolgi-infected monkey model is performed at the time of blood-stage patency (starting at day 11 p.i.). Hypnozoites at days 0 to 6 in the *in vitro* assay may have a drug sensitivity profile different from that of truly dormant hypnozoites that are present in the liver at the time when the animals are treated. Thus, targeting of the truly dormant hypnozoites *in vitro* will be critical in identifying new radical curative drug candidates. To achieve this, we are currently developing a modified assay format whereby P. cynomolgi-infected hepatocytes are treated with test compounds at later time points. Additional studies on parasite liver stages, including in-depth biological analysis of hypnozoites, e.g., by transcriptome sequencing (RNA-seq) analysis of sorted parasites (15), will be instrumental in determining the optimal conditions for an *in vitro* assay that is predictive of radical-cure activity *in vivo*.

## Supplementary Material

Supplemental material
