# Supplementary material for: PI4 Kinase Is a Prophylactic but Not Radical Curative Target in Plasmodium vivax-Type Malaria Parasites
Source: Antimicrob Agents Chemother. 2016 Apr 22;60(5):2858–63. doi: 10.1128/AAC.03080-15 (PMC4862498; doi:10.1128/AAC.03080-15)
Supplement: Supplemental material [file supp_60_5_2858__index.html]

Supplemental material 

# PI4 Kinase Is a Prophylactic but Not Radical Curative Target in Plasmodium vivax-Type Malaria Parasites

## Supplemental material

- Supplemental file 1 -

  Supplemental Tables S1 to S6 and Figures S1 and S2.

  PDF, 247K
