# Supplementary material for: PI4 Kinase Is a Prophylactic but Not Radical Curative Target in Plasmodium vivax-Type Malaria Parasites
Source: Antimicrob Agents Chemother. 2016 Apr 22;60(5):2858–63. doi: 10.1128/AAC.03080-15 (PMC4862498; doi:10.1128/AAC.03080-15)
Supplement: Supplemental material [file AAC.03080-15_zac005165128so1.pdf]

PI4K is a prophylactic, but not radical curative target in *Plasmodium vivax*-type malaria parasites

Anne-Marie Zeeman[a], Suresh B. Lakshminarayana[b], Nicole van der Werff[a], Els J. Klooster[a], Annemarie Voorberg-van der Wel[a], Ravinder R. Kondreddi[b], Christophe Bodenreider [b], Oliver Simon[b], Robert Sauerwein[c], Bryan K. S. Yeung[b], Thierry Diagana[b#], Clemens H.M. Kocken[a#]

Supplementary on-line material

Contents:

1. Methods and materials section: 2

Power calculation for the number of animals needed for the study 2

***Table S1**: power calculation* 2

P. cynomolgi sporozoite production 3

In vivo monkey infection and treatment procedures 3

***Table S2:** PK and clinical blood sampling schedule in monkeys.* 4

Clinical chemistry and hematology 5

In vivo pharmacokinetic (PK) studies of KDU691 and LMV599 in CD-1 mice and monkeys 5

Extraction and quantitation of KDU691 from DBS monkey samples 6

Extraction and quantitation of LMV599 from monkey samples 6

Pharmacokinetic analysis 7

PK-PD relationship analysis 7

***Table S3:** Monkey stratification for the in vivo testing of KDU691* 7

***Table S4**: Monkey stratification for the in vivo testing of LMV 599* 8

2. Results section 8

***Table S5**: Monkey weight during dosing* 8

***Table S6:** Clinical chemistry parameters that in the 599-RC group were outside the normal range at d5* 9

***Figure S1**: Kaplan-Meyer survival curves (in days to patency) for KDU691 in vivo experiment* 9

***Figure S2:** Kaplan Meyer survival curves (in days p.i. to patency) in the LMV599 and PQ treated animals* 10

1. Methods and materials

Power calculation for the number of animals needed for the study

Historical data were taken from rhesus macaques infected with *P. cynomolgi* sporozoites that were drug cured with chloroquine on days 12, 13 and 14 to kill the primary blood stage parasites and monitored for relapse. This showed that survival times (in this case: time to first relapse parasitemia) can be best approximated by the use of a Gamma distribution with the Shape parameter set to two and the Scale parameter set to two.

In order to estimate the power of a number of hypothetical outcomes, the experiment was simulated a 1,000 times using a Gamma distribution for the control and treatment groups employing different group and effect sizes (Table S1). It was calculated that with a group size of four animals any delay to relapse of seven days or more can be detected with more than 80% power.

For the prophylaxis/PK experiment with LMV599, we used three animals and no control group. We based the power calculation on 15 historical control animals, which became patent between days 8-12 and relapsed between days 23-35.

If the three treated animals would not become patent before day 35, the experimental set-up has a statistical power of 99% with a type I error of 0.05. We were only interested in absence/presence of blood stage parasites. If blood stage parasites were detected the single dose prophylaxis has failed; timing is not so important, except that we could see whether the compound has differential activity between liver schizonts (when active, not positive before day 23) and liver hypnozoites (positive after day 23 when not active).

Table S1: *Estimated statistical power for the in vivo evaluation of anti-relapse compounds, from 1,000 simulations using the specified effect size and numbers per group. The table shows that with a group size of 4 animals any delay to relapse of 7 days or more can be detected with more than 80% power (bold).*

	Effect Size (days)				
N	5	6	7	8	9
4	0.597	0.737	**0.813**	0.882	0.913
5	0.620	0.743	0.823	0.887	0.919
6	0.620	0.805	0.862	0.935	0.942

P. cynomolgi sporozoite production

A monkey was infected with 10^6 *P. cynomolgi M* strain blood stage parasites, thawed from a first passage stock (i.e. blood stage parasites from a monkey infected with *P. cynomolgi* M strain sporozoites). For each batch of sporozoites needed, a single monkey was infected with *P. cynomolgi* blood stage parasites. At day 11 and 12 post infection 9 mL infected blood was collected into a Lithium-heparinised tube. The blood was kept at 38°C and fed approximately 500 three to five days old female *Anopheles stephensi* mosquitoes Sind-Kasur strain Nijmegen (Nijmegen University Medical Centre St Radboud, Department of Medical Microbiology) using a glass feeder system as described by Ponnudurai *et al.* [1]. After the second bleed, the monkey was cured by three daily intramuscular injections of 7.5 mg/kg chloroquine [2], monitored for parasite clearance 24 days after chloroquine treatment and returned to colony.

Mosquitoes were kept in climate chambers at 25 °C and 80% humidity and were fed 5% D-glucose on cotton pads daily. Seven days after feeding on the infected rhesus blood, oocyst numbers were determined in midguts of ten individuals, using 1% mercurochrome stain. Mosquitoes were given a second (uninfected) blood meal immediately afterwards to promote sporozoite maturation and invasion of the salivary glands. Sporozoites were isolated from the *P. cynomolgi* infected mosquitoes around day16 after the infected blood meal.

In vivo monkey infection and treatment procedures

Sporozoites were isolated from the *P. cynomolgi* infected mosquitoes around day16 after the infected blood meal. For this, mosquitoes were collected in a beaker and killed with 70% ethanol. After four washes in Leibovitz' L15 medium containing 3% FCS and 2% pen/strep, salivary glands were dissected. Salivary glands were kept on ice at all times, sporozoites were liberated by pottering. The sporozoites were washed with PBS, counted in a Burker-Turk chamber, and diluted to 100,000 spz/mL in PBS. Aliquots of 1 mL were prepared and injected into monkeys via intravenous (i.v.) injection.

For the KDU691 experiment we had a low dose treatment group (691-RC-low), a high-dose treatment group (691-RC-high) and a control group (691-C)

KDU691 was formulated as a micro suspension in 0.5% Methylcellulose/0.5% Tween 80 in water, maximum one hour before dosing. The amount of KDU691 sufficient to treat all animals of one group was weighed, a microsuspension of 10 mg/mL in methylcellulose/tween was made and sonicated (Soniprep 150, Sanyo) with an amplitude of 12-15 µm for 15 min. The dose for each monkey was transferred to a 50 mL tube, which was vortexed extensively just before administration.

To collect samples for KDU691 PK analysis in monkeys, approximately 100 µL blood was taken from the calf vein for each time point and immediately spotted onto DBS filters (PKI Bioanalysis cards, Perkin Elmer). Samples were dried at room temperature for at least two hours and packed in airtight bags containing small desiccant-bags and stored at room temperature until analysis.

Because of genotox issues in mice and rats, a single dose safety/PK/prophylaxis study preceded the radical cure efficacy experiment with LMV599. Three spz-infected monkeys were treated with a single dose of 25 mg/kg LMV599 in 5.6%(w/w) Tween 80/ 44.4% HPMC E3/ 38.9%Soluplus/ 11.1%VitE TPGS solid dispersion after three minutes sonification.

LMV599 radical cure activity was tested in three groups, including a control (599-C), the radical cure group (599-RC) and a short primaquine treatment group (PQ-RC).

When the first relapse was observed, monkeys of the KDU691 experiment were radically cured with primaquine (PQ)/CQ (oral administration of 1.8 mg/kg PQ for 14 days together with i.m. administration of 7.5 mg/kg CQ for the first five days of PQ treatment) to cure both blood stage and liver stage parasites, including hypnozoites.

The monkeys of the LMV599/PQ experiment were cured with a five day CQ treatment (7.5 mg/kg, i.m. administration) at the first relapse and were monitored until the second relapse after which they were cured with CQ (7.5 mg/kg, intramuscular (i.m.) for five days) plus PQ (1.8 mg/kg for 14 days).

***Table S2**: PK and clinical blood sampling schedule in monkeys.*

Groups are named 691 for KDU691 and 599 for LMV599 treatment groups, followed by -C for control group (no compound), -proph for prophylactic treatment (treated immediately after infection), and -RC for radical cure treatment (treated when blood stage positive).

After the fourth day of dosing a yellow discoloration was observed in the 691-proph monkeys, particularly the face and chest areas were very yellow. Therefore the 691-RC group (treated from d11 to d15 pi) was monitored by daily clinical chemistry analysis during dosing to be compared with untreated 691-C.

In the in vivo study with LMV599, a more extensive safety profile was required, hence the clinical sampling of the single dose prophylaxis group at day 0, 1, 7 and 100. The lack of aberrant clinical parameters indicated that the compound was safe to use in a longer dosing regime of five days (d12-16) as radical cure compound, during which also clinical sampling was performed daily.

*[a]: only clinical chemistry samples were taken. *: t = 24 sample was taken only on the 1st day of dosing.*

**Group name**	**Clin. chem Hematology**	**PK sampling**	**PK time points (h post dosing)**
691-C	Daily[a]		
691-proph		1st and 5th day of dosing	t = 0, 1, 4, 6 and 24*
691-RC	Daily[a]	1st and 5th day of dosing	t = 0, 1, 4, 6 and 24*
599-C	Daily		
599-proph	D 0,1,7, 100	day of dosing	t = 0, 1, 2, 4, 8 and 24
599-RC	Daily	1st and 4th day of dosing	t = 0, 1, 2, 4 and 24

Clinical chemistry and hematology

For the clinical analysis serum from 1 mL of clotted blood was analysed using the Cobas Integra 400 plus fully automated chemistry analyser. The following clinical parameters were determined: Alkaline phosphatase; Alanine Aminotransferase; Albumin: Aspartate aminotransfeasr; Bilirubin; Calcium; Cholesterol; Creatine kinase; Creatinine; C-reactive protein: Iron; Phosphate; Gamma-glutamyl transpeptidase; Glucose; Hemoglobin; Potassium; Lactate dehydrogenase; Sodium; Total protein; Urea

Haematological parameters were determined from 1 mL EDTA-blood with a Sysmex XT-2000iV automated system. The following hematological parameters were determined: Basophilic granulocytes; Eosinophilic granulocytes; Erythrocytes; Hematocrit; Leucocytes; Leucocyte differentiation; Lymphocytes; Mean corpusculair hemoglobin; Mean corpusculair hemoglobin concentration; Mean corpusculair volume; Neutrophilic granulocytes; Red cell distribution width; Thrombocytes

In vivo pharmacokinetic (PK) studies of KDU691 and LMV599 in CD-1 mice and monkeys

For *in vivo* PK studies, female CD-1 mice (25-30g) were obtained from the Biological Resource Center, Biopolis, Singapore and were randomly assigned to cages. Mice were allowed to acclimate before initiation of the experiments. Feed and water were given *ad libitum*. Compounds were formulated at concentrations of 2.5 mg/mL and 0.25 mg/mL for a dose of 25 mg/kg and 2.5 mg/kg respectively. The suspension formulation for p.o. dosing contained 0.5% Methyl cellulose and 0.5% Tween 80 in water. After oral dosing, blood and liver samples from mice were collected at 0.08 to 24 h post dosing. Groups of three mice were used for each time point. Blood was centrifuged at 13,000 rpm for 7 min at 4 °C, plasma harvested and stored at -20 °C until analysis. Liver tissue samples were excised, dipped in PBS, gently blotted with absorbent paper, dried, weighed and stored at -20 ºC until further analysis.

For LC/MS/MS analysis, 50 µL of plasma samples were precipitated using 180 µL of acetonitrile:methanol:acetic acid (90:9.8:0.2) containing either related compound or warfarin as internal standard. After vortexing and centrifuging the mixture, the supernatant was removed and 5 µL of sample analyzed. Whole liver tissue was homogenized in 2 mL of PBS. 50 µL of liver homogenate was taken and processed as described above for plasma samples. The standard calibration curve was prepared by spiking blank plasma and liver tissue with different concentrations of the compound. In addition, quality control samples with three different concentrations were prepared in respective blank matrix and analyzed together with the unknown samples for validation purposes. Analyte quantitation was performed by LC/MS/MS using optimized conditions for each compound.

For KDU691, Liquid chromatography was performed using an Agilent 1100 HPLC system (Santa Clara, CA), with the Agilent Zorbax XDB Phenyl (3.5µ, 4.6x75 mm) column at an oven temperature of 45 ºC, coupled with a API4000 triple quadruple mass spectrometer (Sciex Applied Biosystems, Foster City, CA). Instrument control and data acquisition were performed using Applied Biosystems software Analyst 1.4.2. The mobile phases used were A: water-acetic acid (99.8:0.2, v/v) and B : acetonitrile-formic acid (99.9:0.1, v/v), using a gradient 0 – 0.5 min (35 % B), 0.5 – 2.0 min (35 – 85 % B), 2.0 – 3.92 min (85 % B), 3.92 – 4.0 min (85 – 35 % B) and 4.0 – 4.75 min (35 % B), with a flow rate of 1.0 mL/min, and a run time of 4.75 min. Under these conditions the retention time was 2.22 minutes. Multiple reaction monitoring (MRM) was combined with optimized mass spectrometry parameters to maximize detection specificity and sensitivity. The most intense MRM transition (420.2/269) was used for quantitation using electrospray ionization in the positive mode. The recovery of the compound from both plasma and liver tissue were good and consistent across the concentration range studied. The lower limit of quantification was 3.0 ng/mL in plasma and 3.2 ng/g in liver. For LMV599, the analysis was similar as monkey samples.

Extraction and quantitation of KDU691 from DBS monkey samples

Two-1/8" (~3 mm) dried blood spot punches of blank (for control samples and zero samples), calibration standards, QCs, and unknown samples were added into the appropriate tube in a 96-well format. A 25 µL aliquot of the internal standard working solution (200 ng/mL of [M+8] KDU691 in 90:10 water:acetonitrile, v:v,) was added into all the tubes except those for the control blanks, to which a 25 µL aliquot of 90:10 water:acetonitrile, v:v, was added. A 500 µL aliquot of methanol was added to each tube and the plate was sonicated for approximately 10 minutes followed by vortexing for approximately 5 minutes on a pulse-vortex mixer at a speed setting of about 50. The plate was centrifuged at about 3000 × g for about 10 minutes at approximately 10 °C. Using a Quadra 96 TomTec system, the resulting supernatant was transferred into the corresponding well of a clean 1-mL 96-well plate, followed by evaporation to dryness under a flow of nitrogen at about 45 °C. The sample residues were reconstituted with 150 µL of 10% acetonitrile in water (v/v) containing 0.2% formic acid. The plate was mixed well and centrifuged at about 750 × g for about 5 min at approximately 10 °C. A 20 µL aliquot of the reconstituted sample extract was injected onto the LC-MS/MS system. Analyte quantitation was performed by high performance liquid chromatography coupled with tandem mass spectrometry (LC/MS/MS). Liquid chromatography was performed using an Shimadzu HPLC system, with the Waters XSelect C_{18} (2.5 µm, 50 x 4.6 mm) column at oven temperatures of 60 °C, coupled with a Sciex API4000 triple quadruple mass spectrometry (Sciex Applied Biosystems, Concord, Ontario, Canada). The mobile phases used were A: water-formic acid (99.9:0.1, v/v) and B: acetonitrile (100%), using a gradient 0.01 – 2.5 min (35% B), 2.5 – 2.6 min (35 – 95% B), 2.6 – 3.0 min (95% B), 3.0 – 3.1 min (95 – 35 % B) and 3.1 – 5 min (35% B), with flow rate of 1.0 mL/min, and run time of 5 min. Multiple reaction monitoring (MRM) was combined with optimized MS parameters to maximize detection specificity and sensitivity. The most intense MRM transitions (420.2/279) was used for quantitation using electrospray ionization in the positive mode. The recovery of the compound from blood was good and consistent across the concentration range studied. The lower limit of quantification was 20 ng/mL using two-1/8" (~3 mm) dried blood spot punches expressed as base. Calibration curve and quality control samples was prepared freshly and analyzed with study samples. The results were accepted if relative standard deviation was less than 15%.

Extraction and quantitation of LMV599 from monkey samples

Plasma samples were processed by protein precipitation using acetonitrile:methanol:acetic acid (89.8:10:0.2) to recover both analytes and internal standard (100 ng/mL of warfarin) using a 8 to 1 extractant to plasma ratio. After vortex mixing and centrifuging the mixture, the supernatant was removed and 5 µL of sample analyzed. Analyte quantitation was performed by high performance liquid chromatography coupled with tandem mass spectrometry (LC/MS/MS). Liquid chromatography was performed using an Agilent 1260 HPLC system (Santa Clara, CA), with the Kinetex Phenyl Hexyl (2.6 µm, 4.6 x 50 mm) column at oven temperatures of 45 °C, coupled with a QTRAP5500 triple quadruple mass spectrometry (Sciex Applied Biosystems, Foster City, CA). Instrument control and data acquisition were performed using Applied Biosystems software Analyst 1.6.2. The mobile phases used were A: water-acetonitrile-acetic acid (94:5:1, v/v) and B: acetonitrile-acetic acid (99:1, v/v), using a gradient 0 – 0.7 minutes (10% B), 0.7 – 2.7 minutes (10 – 90% B), 2.7 – 3.7 minutes (90% B), 3.7 – 4.2 min (90 – 10% B) and 4.2 – 5 minutes (10% B), with flow rate of 1.0 mL/min, and run time of 5 minutes. Multiple reaction monitoring (MRM) was combined with optimized MS parameters to maximize detection specificity and sensitivity. The most intense MRM transition (425.2/394.1) was used for quantitation using electrospray ionization in the positive mode. The recovery of the compounds from plasma was good and consistent across the concentration range studied. The lower limit of quantification was 0.15 ng/mL in plasma. Calibration curve and quality control samples (three concentration levels in triplicate) was prepared freshly and analyzed with study samples. The results were accepted if relative standard deviation was less than 15%.

Pharmacokinetic analysis.

Mean values of compound concentrations in plasma and liver were obtained from three animals at each time point and plotted against time to generate concentration-time profiles. Pharmacokinetic parameters were determined using WinNonlin Professional, version 5.0.1 (Pharsight, California, USA), by non-compartmental modeling using software model 200 for oral dosing. The oral bioavailability (F) was calculated as the ratio between the area under the curve from 0 to infinity (AUC_{inf}) following oral administration and the AUC_{inf} following intravenous administration corrected for dose ($F = AUC_{p.o.} * dose_{i.v.} / AUC_{i.v.} * dose_{p.o.}$).

PK-PD relationship analysis.

The compound concentration that inhibited 50% of *P. cynomolgi* growth (IC_{50}) was used to calculate the threshold (TRE = 3*IC_{50}) and the PK-PD indices. The C_{max}/TRE was defined as the ratio of peak plasma concentration (C_{max}) to the threshold (3*IC_{50}), the $AUC_{0\text{-}24}$/TRE was defined as the ratio of area under the curve from 0 to 24 hours ($AUC_{0\text{-}24}$) to the threshold, and the percent time over threshold (%*T>TRE*) was defined as the percentage of the 24 hour period during which the compound concentration exceeded the threshold.

Table S3: *Monkey stratification for the in vivo testing of KDU691.*

Monkeys were stratified according to gender, age and weight. In the KDU691 experiment each group contained two males and two females.

Treatment group	Animal ID	Date of birth	Weight at infection (kg)	Sex
691-C	R06087	10-07-06	8.94	M
	R06088	10-07-06	10.62	M
	Ri0511022	01-11-05	5.83	F
	Ri0511130	04-11-05	5.43	F
691-proph	R06021	17-05-06	7.26	M
	R06052	13-06-06	6.88	M
	R01066	13-07-01	5.97	F
	R02056	17-06-02	7.72	F
691-RC	R07002	13-04-07	8.94	M
	R07035	25-05-07	7.75	M
	Ri0511008	01-11-05	6.78	F
	Ri0511134	05-11-05	6.44	F

Table S4: *Monkey stratification for the in vivo testing of LMV 599.*

Monkeys were stratified according to age, gender and weight. Each group contained at least one male.

Treatment group	Animal ID	Date of birth	Weight at infection (kg)	Sex (F/M)
599-C	R00065	02-09-00	10.26	F
	R10082	11-06-10	5.53	F
	R09106	14-06-09	7.19	M
	R09157	16-08-09	7.54	M
599-RC	R10078	10-06-10	5.50	F
	R06044	06-06-06	5.92	F
	R09038	02-05-09	5.91	F
	R10119	09-07-10	6.44	M
PQ-RC	R10064	04-06-10	7.46	F
	R05013	02-05-05	6.60	F
	R05063	27-06-05	6.56	F
	R10103	24-06-10	6.51	M

2. Results

Table S5*: Monkey weight during dosing.*

Average weight of each treatment group (in kg) at the time of the first drug treatment (d0) and the weight change (in % of weight at d0) during the treatment. Monkeys in the control group of the KDU691 in vivo testing lost on 5.2% of their body weight due to the daily sedations and gastric feeds, the monkeys in the treatment groups also had a weight loss of 3.5% of the initial weight (averaged over the three treatment groups).

The LMV599 control monkeys lost 2.7% of the bodyweight when compared to the weight at the beginning of the drug dosing. The monkeys in the LMV599 treatment group lost 3.9% of the initial weight. In the monkeys that received primaquine treatment, a mean weight loss of 2.9% was observed.

Treatment group	Avg weight at first dose (kg) d0	Weight change d1 (%)	Weight change d2 (%)	Weight change d3 (%)	Weight change d4 (%)
691-C	7.92±3.01	-2.7	-3.2	-4.6	-5.2
691-proph	6.64±0.88	-0.4	-2.4	-2.7	-3.2
691-RC	7.44±1.37	-0.9	-1.3	-2.9	-2.9
599-C	7.67±1.89	-1.1	-1.2	-2.1	-2.7
599-RC	5.82±1.25	-2.1	-1.5	-2.9	-3.9
PQ-RC	6.65±0.51	-0.8	-1.2	-2.6	-2.9

Table S6: *Clinical chemistry parameters that in the 599-RC group were outside the normal range at d5.*

All of the deviant clinical parameters are liver enzymes, indicating that LMV599 seems to harm the liver. The parameters were compared between control and LMV599-treated animals. The average of each group ±SD is shown here. P-values were calculated and indicate that alkaline transaminase and gamma-glutamyl transferase (bold) were significantly different between the groups.

Clinical chem. parameter	Control	599-RC	p-value
Alkaline phosphatase	195±83	545±289	0.0788
**Alkaline transaminase**	68±28	404±184	**0.0269**
Aspartate transaminase	84±46	220±89	0.0628
**Gamma glutamyl transpeptidase**	46±12	237±156	**0.0409**

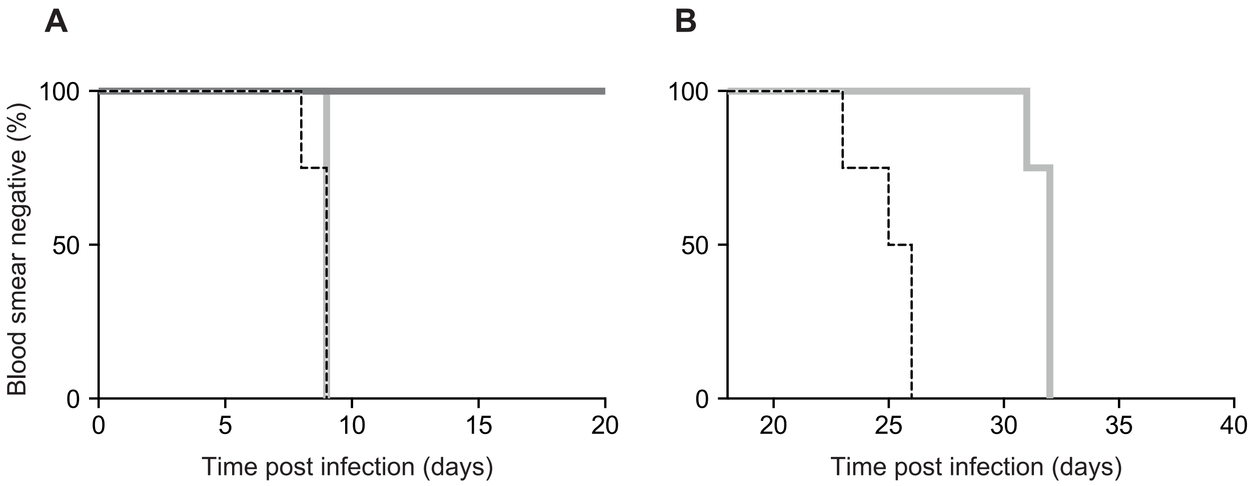

Figure S1: Kaplan Meyer survival curves (in days to patency) of KDU691 *in vivo* experiment.

Three groups of four animals were injected with 100,000 *P. cynomolgi* sporozoites (i.v). Groups were named 691 (KDU691-experiment), followed by -C for control group (no compound), -proph for prophylactic treatment, and -RC for radical cure treatment.

A) Days to patency of 691-proph (dark grey line), 691-C (black dotted line) and 691-RC (light grey line) groups. None of the 691-proph animals became blood stage positive during the 102 days of follow-up. All other animals were blood stage positive at day 9 p.i. and treatment was started at day 11 p.i. B) Days to first relapse. Animals were monitored for presence of blood stage parasites by thin film smears from day 22 onward. The 691-C monkeys relapsed 25 ± 1.4 days p.i. (black dotted line). Animals in the 691-RC group (light grey line) relapsed 31.8 ± 0.5 days p.i.

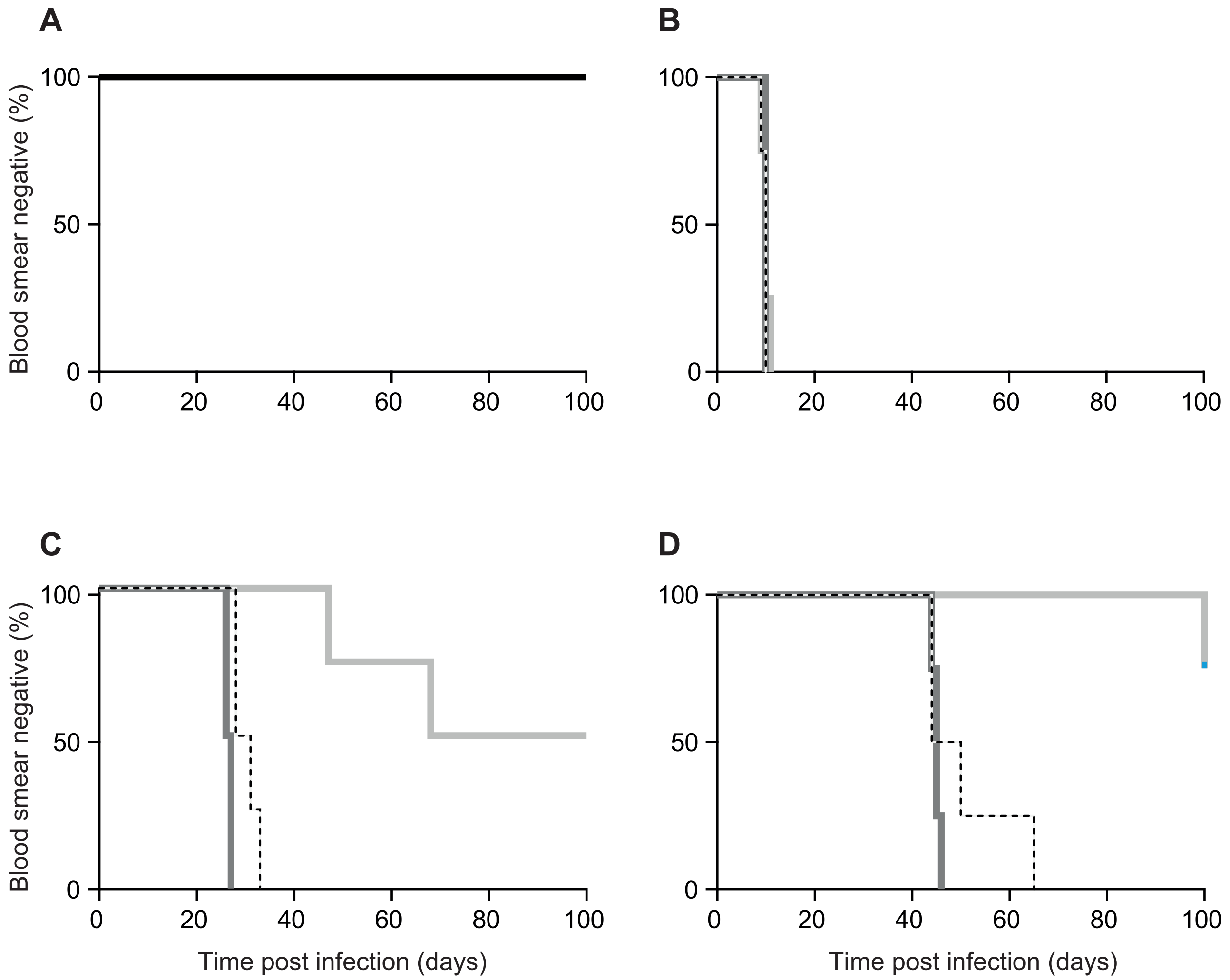

Figure S2: Kaplan Meyer survival curves (in days p.i. to patency) in the LMV599 and PQ treated animals:

A) Three animals were infected with 100,000 spz (i.v.), followed by a single oral dose of 25 mg/kg LMV599 (599-proph group). During the 100 days of monitoring of the 599-proph animals none became blood stage positive (black line); B) Days p.i. to patency of the radical cure groups. Twelve animals were infected with 100,000 *P. cynomolgi* spz each on day 0. All animals became blood stage positive between day 9 and 11 p.i. and were treated from day 12-16 p.i. with 5 i.m. doses of CQ in combination with oral application of either empty vehicle (599-C, black dotted line), 25 mg/kg LMV599 (599-RC, dark grey line), or 1.8 mg/kg PQ (PQ-RC light grey line); C) Days p.i. to the first relapse. After the treatment, absence of blood stage parasites was confirmed. From day 23 p.i. onward, the animals were monitored for the reappearance of blood stage parasites. 599-C animals (black dotted line) relapsed on day 30 ± 2.5 p.i., the 599-RC arm (dark grey line) became blood stage positive again on day 26.5 ± 0.6 p.i. The animals that received 5 day PQ treatment (light grey line) relapsed on day 57.5 ± 14.8 p.i (2 out of 4). At the first relapse, animals received the second five-day CQ-treatment, and were monitored until blood stage positive or days 100 p.i.; D) Days p.i. to the second relapse. The 599-C group monkeys relapsed for the second time on day 51 ± 15 (black dotted line), the 599-RC treatment group on day 45 ± 0.8 (dark grey line). In the short PQ-RC treatment group, one monkey had a second relapse at day 100 p.i. (light grey line)

References

1. Ponnudurai T, Lensen AH, Van Gemert GJ, Bensink MP, Bolmer M, Meuwissen JH: **Infectivity of cultured Plasmodium falciparum gametocytes to mosquitoes.** *Parasitology* 1989, **98 Pt 2:**165-173.
2. Mendes C, Dias F, Figueiredo J, Mora VG, Cano J, de Sousa B, do Rosario VE, Benito A, Berzosa P, Arez AP: **Duffy Negative Antigen Is No Longer a Barrier to Plasmodium vivax - Molecular Evidences from the African West Coast (Angola and Equatorial Guinea).** *PLoS Negl Trop Dis* 2011, **5:**e1192.
